# The Fate of Foodborne Pathogens in Manure Treated Soil

**DOI:** 10.3389/fmicb.2021.781357

**Published:** 2021-12-10

**Authors:** Zoe Black, Igori Balta, Lisa Black, Patrick J. Naughton, James S. G. Dooley, Nicolae Corcionivoschi

**Affiliations:** ^1^Grassland and Plant Sciences Branch, AFBI Crossnacreevy, Sustainable Agri-Food Sciences Division, Agri-Food and Biosciences Institute, Belfast, United Kingdom; ^2^Bacteriology Branch, Veterinary Sciences Division, Agri-Food and Biosciences Institute, Belfast, United Kingdom; ^3^Nutrition Innovation Centre for Food and Health (NICHE), School of Biomedical Sciences, Ulster University, Coleraine, United Kingdom; ^4^Faculty of Bioengineering of Animal Resources, Banat University of Animal Sciences and Veterinary Medicine, King Michael I of Romania, Timisoara, Romania

**Keywords:** manure, bacterial pathogens, soil, agriculture, pathogen persistence, zoonotic pathogens

## Abstract

The aim of this review was to provide an update on the complex relationship between manure application, altered pathogen levels and antibiotic resistance. This is necessary to protect health and improve the sustainability of this major farming practice in agricultural systems based on high levels of manure production. It is important to consider soil health in relation to environment and land management practices in the context of the soil microflora and the introduction of pathogens on the health of the soil microbiome. Viable pathogens in manure spread on agricultural land may be distributed by leaching, surface run-off, water source contamination and contaminated crop removal. Thus it is important to understand how multiple pathogens can persist in manures and on soil at farm-scale and how crops produced under these conditions could be a potential transfer route for zoonotic pathogens. The management of pathogen load within livestock manure is a potential mechanism for the reduction and prevention of outbreaks infection with *Escherichia coli*, *Listeria Salmonella*, and *Campylobacter*. The ability of *Campylobacter*, *E. coli*, Listeria and *Salmonella* to combat environmental stress coupled with their survival on food crops and vegetables post-harvest emphasizes the need for further study of these pathogens along with the emerging pathogen *Providencia* given its link to disease in the immunocompromised and its’ high levels of antibiotic resistance. The management of pathogen load within livestock manure has been widely recognized as a potential mechanism for the reduction and prevention of outbreaks infection but any studies undertaken should be considered as region specific due to the variable nature of the factors influencing pathogen content and survival in manures and soil. Mediocre soils that require nutrients could be one template for research on manure inputs and their influence on soil health and on pathogen survival on grassland and in food crops.

## Introduction

With the global population continuing to rise, there is an ever-increasing pressure placed on soil to support the intensification of agriculture (Waggoner, 1995; Tilman et al., 2002; Tomley and Shirley, 2009; Blaiotta et al., 2016; Manyi-Loh et al., 2016; Struik and Kuyper, 2017; Chaudhari et al., 2021). Agricultural demands vary from primary food source crop production to production of fodder crops and direct grazing used in livestock production. This demand, combined with a migration toward monoculture crop production systems, results in increasing amounts of manure, essentially a waste product, produced by livestock (Aarons et al., 2009; Plourde et al., 2013; Attwood et al., 2019). However, manures are now considered as organic fertilizer which is an essential resource to aid the intensification of agricultural production (McLaughlin et al., 2011; Mazur and Mazur, 2015; Chaudhari et al., 2021). Manures are routinely applied to grassland and arable sites, with multiple studies indicating an array of benefits such as provision of essential nutrients (e.g., potassium, phosphorous, nitrogen) for plant growth, increased soil carbon content, greater carbon sequestration and neutralization of soil acidity (Boateng et al., 2006; Risse et al., 2006; Zhang and Fang, 2007; Wortmann and Shapiro, 2008; Loss et al., 2019; Ekman et al., 2021). A study by Anwar et al. (2005) highlighted the benefits of manure utilization in tandem with inorganic fertilizers as an approach to sustainably improve both soil productivity and quality. Increasing reliance on manure as a soil enhancer, coupled with the need to dispose of snowballing waste production, has raised many questions regarding the safe use of manures to achieve and maintain sustainable agriculture (Pratt, 1979; Adegoke et al., 2016; Manyi-Loh et al., 2016). Sustainable agriculture is defined as agricultural practices which can “meet current and future societal needs, for ecosystem services, and for healthy lives” (Tilman et al., 2002) and relies on the safe use of manures from a soil, crop, environment and human consumption perspective. The idea of Planetary Health identifies a link between human health, biodiversity and ecosystems, where nutrients and organisms introduced to the soil microbiome interact with the associated ecosystems (Zhu et al., 2019). The interaction with the associated ecosystems is dependent on human activity as it could place soil at the center of antimicrobial resistance spread due to the presence of pollutants which can include antimicrobials and antimicrobial resistant pathogens. The spread of animal manure as a soil amendment (Zeng et al., 2017) intensifies the spread of antimicrobial resistance genes (ARGs) as they are prominent in the animal gut due to the overuse of antibiotics in farming or due to the intensive use of in-feed antibiotics (Zhao Q. et al., 2018). This evidence clearly requires an understanding of the role of soil aligned with the use of manure to understand the dissemination of ARGs and pathogens within the environment.

Agriculture in Northern Ireland (NI) has changed considerably in the past 80 years with expanding livestock sectors, such as cattle and sheep production, resulting in a shift toward a predominantly grassland agriculture. Approximately 93% of the total area farmed in NI is grassland (EARA, 2020). Livestock farming is dominant in NI having doubled between 1940 and 2016 (EARA, 2020). Northern Ireland produces approximately 13 and 25%, respectively, of the total manure output and total slurry output across the United Kingdom (UK) (Smith and Williams, 2016). This contribution of undiluted slurry production is high as the relative total land area of NI is approximately 5% that of the rest of the United Kingdom. The high ratio of slurry produced to area farmed, in tandem with slurry restrictions such as application limits of 30–50 m^3^ per hectare in one application, results in the need to control and balance the spreading of slurry on land as there is potential for excessive applications and misuse. In addition to cattle slurry, the NI livestock sector produces pig and poultry manures and additional manures in the form of digestates are produced from an increasing number of anaerobic digesters, which are often fed pig, poultry, and cattle waste (EARA, 2020). The Sustainable Agricultural Land and Management Strategy (SALMS) report (EARA, 2020) advises that the performance of NI soils is, mediocre with only 18% of analyzed soils at optimum fertility and only 36% of soils were reported as optimal pH for grass production. These low statistics for optimum soil health from a sector of high economic importance suggests NI soil systems could be a good template for research with regards to the influence of agricultural practices on soil health, particularly on grassland farming in a temperate setting. The heavy reliance on the livestock industry, increasing shifts toward intensive production systems (e.g., zero grazing systems) along with large livestock/area farmed ratios makes NI a key example of the potential influence of spreading of manure onto agricultural land. There is a possibility of designing and implementing changes with the aim of improving these statistics as soil health relationships are context-dependant (Yang et al., 2020).

The aim of this review was to provide an update on the complex relationship between manure application and altered pathogen levels. This is necessary to protect health and improve the sustainability of this major farming practice in the context of Northern Ireland, a useful model system for agricultural systems based high levels of manure production.

Soil health has been recognized as the ability of the soil to cope with anthropogenic factors associated with agricultural production, whilst maintaining the provision of ecosystem services (Kibblewhite et al., 2008; Larney et al., 2016). According to Kibblewhite et al. (2008), soil health is reliant on nutrient cycling, soil structure, transmission of carbon and pest/disease regulation (Kibblewhite et al., 2008). It is important to consider soil health in relation to environmental and land management practices. As soil fertility encompasses chemical, physical and biological properties, it can be considered a measure of soil health (Kibblewhite et al., 2008). Agricultural practices impact soil health in various ways (Bore et al., 2017; see Table 1). As common agricultural practices have the ability to influence these values, it is important to consider their role in achieving optimum production capacity from farmland. The application of fertilizers can have an indirect influence on soil bacterial communities by first altering the pH (Zhang et al., 2017). Changes in pH have been shown to alter the structure of the bacterial community present, and therefore have the potential to reduce soil health through knock on effects of reduction of soil services such as nutrient cycling (Zhang et al., 2017).

**TABLE 1:** Examples of the impact of common agricultural practices on soil health.

Common practices	Example of impact on soil health / fertility	References
Monoculture cropping Over grazing Tillage	Reduced and/or altered biological diversity	Mikha and Rice, 2004; Zhao Y. et al., 2018; Salaheen and Biswas, 2019; Yang et al., 2020
	Reduced organic carbon content	
	Soil nutrient depletion	
	Decreased pH levels	
	Soil structural change	
Addition of artificial fertilizer Addition of manure	Altered nutrient availability	Mikha and Rice, 2004; Bünemann et al., 2006
	Altered pH levels (e.g., soil acidification by added N)	
	Altered biological diversity	
	Soil structural change	

To date, very little work has been carried out on the persistence of multiple pathogens in manures and on soil at farm-scale and the impact of both long and short term use of these manures on the soil microbial dynamics is not well understood (Wimalarathna et al., 2013; Li et al., 2017). Crops produced under these conditions have the potential to contain organisms found within the soil microbiome, therefore creating a potential route of transfer of pathogens into the food chain causing illness (Tilman et al., 2002; Larramendy and Soloneski, 2016; García-Sánchez et al., 2018). Some of the major zoonotic bacterial pathogens found in soil and manures are considered herein (Table 2).

**TABLE 2:** Background information for examples of zoonotic pathogens which occur in manure (CFU, Colony Forming Units; VNBC, Viable but non-culturable; STEC, Shiga toxin-producing *Escherichia coli*).

Pathogen	Characteristics	Infective dose CFU	Common agricultural settings	Common pathogenic species	Examples of survival responses to environmental stresses	References
*Campylobacter spp.*	Helical microaerophilic, Gram-negative	500–800	Poultry, cattle, pigs, waterbodies	*C. jejuni*	Biofilm formation VBNC bacterial co-habitation Attachment to biotic and abiotic surfaces through biofilm formation Drug resistance Host invasion	Stanley and Jones, 2003; Murphy et al., 2006; Bronowski et al., 2014; Chlebicz and Śliżewska, 2018; Sibanda et al., 2018
*Escherichia coli*	Rod shaped, facultative anaerobe, Gram-negative.	Extremely low e.g., 10 CFU	Cattle, pigs and sheep, soil, water bodies	STECs e.g., O157		Tuttle et al., 1999; Fremaux et al., 2008; Segura et al., 2018; Capellini et al., 2020
*Salmonella* spp.	Rod shaped, facultative anaerobe, Gram-negative.	Debated, comparatively higher than that of *Campylobacter* and *E. coli*	Pigs, cattle, soil, water bodies	*Salmonella typhimurium*		Waldner et al., 2012; Dar et al., 2017
*Providencia* spp.	Rod shaped, facultative anaerobe, Gram-negative.	Suggested to be high	Soil, water, livestock intestines	*Providencia stuartii*	Multi-drug resistance Biofilm formation	O’Hara et al., 2000; Wie, 2015; El Khatib et al., 2017; Kurmasheva et al., 2018

## Bacteria, Manure, and Soil

Bacteria can survive in varying oxygen concentrations, temperatures, pH levels and moisture contents. Significant correlations between bacterial diversity and annual precipitation and pH levels were found in a study of maize cropping systems in China (Tan et al., 2020). If microorganisms are incapable of coping with changing soil conditions they face competition and removal in the contest for nutrients (Ratzke and Gore, 2018). The newly arrived microorganisms find the new conditions favorable and will survive better than the native communities (Sharma and Reynnells, 2016). Predatory activity by organisms such as protozoans can also influence bacterial survival which is an important regulator of population levels (England et al., 1993).

The microbial populations associated with manure varies due to numerous influential factors, including the producing animal, its feeding regime and the waste management practices (Albihn and Vinnerås, 2007; van Vliet et al., 2007; Jokinen et al., 2012; Lopatto et al., 2019). Manure application can inadvertently spread zoonotic diseases to humans while both animal and plant populations can also be vulnerable to disease (Albihn and Vinnerås, 2007; Spiehs and Goyal, 2007; Longhurst et al., 2019; Tran et al., 2020). In addition to health implications for humans, livestock and crops, bacterial pathogens can also be costly to the economy. Effects encompass the direct costs and food sales effected by microbiological quality and production quantity concerns stemming from poor soil health and outbreaks of illness caused by bacterial pathogens (Swanenburg et al., 2001; Chlebicz and Śliżewska, 2018). Bacteria found in manures, e.g., *E. coli* and *Salmonella*, have often been implicated either directly or indirectly to outbreaks of human illness (Pell, 1997; Bicudo and Goyal, 2003; Guan and Holley, 2003; Hutchison et al., 2004). Viable pathogens contained within manure spread on agricultural land may be distributed by leaching, surface run-off, water source contamination and contaminated crop removal (Fenlon et al., 2000; Albihn and Vinnerås, 2007; Semenov et al., 2009; dikovic-Kolic et al., 2014; Alegbeleye and Sant’Ana, 2020; Mügler et al., 2021). A relationship therefore exists between agricultural practices and subsequent pathogen transfer to multiple ecosystems. For example, transfer of *E. coli* was shown from untreated poultry manure to fresh produce (Atidégla et al., 2016). Protecting food and water supplies from animal fecal contamination is of high importance in an attempt to preserve human health, animal health and agricultural sustainability (Pell, 1997; Kudva et al., 1998; Olson, 2001). Altered products may have a detrimental impact on human and livestock populations, particularly when contamination reaches areas beyond the agricultural land itself (Tilman et al., 2001, 2002; Sharpley et al., 2013; Ekman et al., 2021). There is a relationship between the source of manures and the pathogen content of the manure (Larramendy and Soloneski, 2016). Prevention of zoonosis within livestock populations is essential in order ensure high biosecurity standards (Albihn and Vinnerås, 2007; Layton et al., 2017) and the reduction of pathogen presence in manures will increase their sustainability and safe use in the environment (Barbour et al., 2002; Albihn and Vinnerås, 2007; Jung et al., 2014). Any studies undertaken should be considered as region specific due to the variable nature of the factors influencing pathogen content and survival in manures and soils (e.g., feed regime, climate, soil, and precipitation) (Bradford et al., 2013).

Soil is influenced by manure, with application reported to have positive impacts on the physical and chemical properties associated with soil health and fertility such as increased soil carbon content, water retention and nutrient supplies (Barbour et al., 2002; Wilson et al., 2008; Bradford et al., 2013; Jung et al., 2014). Large applications have the capacity to increase dispersion of soil particles by introducing high saline ion contents (Barbour et al., 2002). The apparent differences in the effect of manure application on soil structure and composition has been attributed to varying climatic conditions. Greater soil leaching removed excess sodium ions from the soil, eliminating the negative impact seen in semi humid and arid soils (Wagenaar et al., 2013). Any changes to soil properties can lead to alterations of biological properties with potential to restructure the soil microbiome. Microorganisms included in manure are introduced to the soil during application (Murphy et al., 2006; van Vliet et al., 2007; Costa and Iraola, 2019). The introduction of foreign microbe populations, and changing environmental conditions (such as pH and salinity) may favor different microbes to the resident population and have the potential to influence the biodiversity and overall structure of the soil microbiome (Wallis, 1994; Kothary and Babu, 2001; Albihn and Vinnerås, 2007; Epps et al., 2013). For example, long term artificial fertilizers containing N, K and P have been shown to reduce soil pH while composted cattle manures have been shown to increase pH, crop yield and microbial activity in a flooded rice cropping system (Bronowski et al., 2014). Manure treated soils were found to have enhanced microbial activity with dominant bacteria such as Proteobacteria preferring high nutrient environments resulting from the breakdown of complex compounds. Soil which received no manure had dominant bacteria such as Acidobacteria, commonly found in nutrient limited soils (Stanley and Jones, 2003). However, Fernandez et al. (2020) found little direct influence of management practices on soil bacteria community structure, attributing variations in diversity to site locations instead (Szott and Friese, 2021). Increased crop yields could be linked to conditions, such as increased pH, providing optimum conditions for bacteria associated with the provision of available nutrients for crop growth, in turn enhancing plant growth.

## Bacterial Pathogens in Manure

Pathogen prevalence within different manures is subject to variables including producing animal species, feeding regimes and antimicrobial additives. Feeding regimes such as increased feed acidity has been proven to reduce *Salmonella* in fattening pigs (Bui et al., 2011). Manure application to soil may assist integration of pathogens with other environments through general pathways such as direct contact with livestock, contact with fresh produce and water bodies (dikovic-Kolic et al., 2014; Attwood et al., 2019; Rukambile et al., 2019). Pathogens react differently to stress and non-optimum conditions e.g., *Listeria* spp., are highly adaptable in low water availability, low pH and low temperature soil environments (Morita et al., 2004). Application of poultry and pig manure to arable lands has the potential to disseminate *E. coli* to soil and beyond (Murphy et al., 2006) and *Salmonella* have been identified in environments with little or no oxygen, e.g., slurry pits, and at low temperatures (Colles et al., 2008). The repetitive nature of modern farming, monoculture crops and similar livestock populations (i.e., hosts) in particular areas, allow pathogens to adapt and specialize in the survival and reinfection of existing host populations (Wesley et al., 2000). The impact of monoculture on bacterial diversity in soils is dependent on crop rotations (An et al., 2018). Pathogens are less likely to thrive in genetically diverse ecosystems which supports the theory that repetitive farming may cause pathogen populations to become established within the farming environment (Harvey et al., 1999).

Bacterial pathogens found within manure and associated with bacterial outbreaks include, but are not limited to, *Campylobacter*, *Salmonella* and strains of pathogenic *E. coli* such as O157:H7 (Swanenburg et al., 2001; Indikova et al., 2015; Manyi-Loh et al., 2016; Zhong et al., 2020). An example of an emerging pathogen found in manure and soil is *Providencia* spp. and it is an important reminder of the need for ongoing surveillance in an area that is likely to experience change as agricultural practices evolve. *Campylobacter* spp.

### *Campylobacter* spp.

*Campylobacter* is one of the most common causes of food-borne diseases in the United States of America and Europe (Murphy et al., 2006; Wilson et al., 2008; Wagenaar et al., 2013; Costa and Iraola, 2019). Humans can develop campylobacteriosis from a low infective dose of only 500–800 CFU (Wallis, 1994; Kothary and Babu, 2001; Epps et al., 2013). and *Campylobacter jejuni* is responsible for most cases of infection (Epps et al., 2013; Costa and Iraola, 2019). *Campylobacter* spp. have been reported in unpasteurized dairy products, untreated water and manures (Stanley and Jones, 2003; Wagenaar et al., 2013; Bronowski et al., 2014; Szott and Friese, 2021) and are prevalent in many food-producing animals including poultry, cattle, sheep and pigs (Szott and Friese, 2021). It has been reported that 100% of *Campylobacter* isolates from poultry and cattle fecal samples are *C. jejuni* while 97% of pig fecal isolates were identified as *C. coli* (Morita et al., 2004; Colles et al., 2008; Bui et al., 2011; Rukambile et al., 2019). Other studies have shown that cattle are common reservoirs of *C. jejuni* and *C*. *lari* (Wesley et al., 2000; An et al., 2018). Harvey et al. (1999) highlights the prevalence of *C. jejuni* in pigs, albeit a lower rate than *C*. *lari*. Morita et al. (2004) determined prevalence of *Campylobacter* spp. in cattle, and pig and poultry fecal samples of 76.0, 63.8, and 50.0%, respectively which could potentially represent a source of contaimination in soils via manure application as *C. jejuni* can adapt to non-ideal environments tolerating higher oxygen levels and reduced nutrient availability (Bronowski et al., 2014). It has been suggested that *Campylobacter* can increase biological diversity, by lowering of oxygen concentrations thereby reducing oxygen stress with *C. jejuni* having been shown to contribute to mixed culture biofilm under aerobic conditions (Zhong et al., 2020). Increased survival success has been demonstrated in cultures containing *Pseudomonadaceae* as *Campylobacter* possess the ability of to attach to their biofilms (Indikova et al., 2015). Their ability to enter the viable but non-culturable state (VBNC) in response to high oxygen levels has been recognized, however, the infectiveness of the VBNC is not well documented (Bronowski et al., 2014; Zhong et al., 2020; Szott and Friese, 2021). Temperature has been suggested as a key factor in the ability of *Campylobacter* to survive in the environment. An investigation by Bui et al. (2011) determined that *C. coli* was more sensitive to aerobic conditions at temperatures of 15^°^C or more compared to temperatures of 4^°^C, suggesting temperature is a key factor in the response to stresses such as oxygen content (Bui et al., 2011). A study by Brandl et al. (2004) demonstrated better *C. jejuni* survival on plant roots and leaves at lower temperatures (10 and 16°C), than at higher temperatures (33 and 37°C). The same study identified a relative increase in survival of *C. jejuni* on wounded plant leaves at 10°C, suggesting potential for amplified survival during harvest and food preparation. It has been suggested that the frequency of campylobacteriosis is due to the prevalence of the organism in different environments, such as meat plants, once shed from animal hosts (Li et al., 2017). *Campylobacter* are thought not to multiply outside of a host due to environmental stresses and lack of survival at oxygen concentrations above 5% (Brandl et al., 2004; Wimalarathna et al., 2013; Li et al., 2017; García-Sánchez et al., 2018; Gundogdu and Wren, 2020).

### Escherichia coli

The facultative anaerobe *E. coli* is diverse with a wide range of pathotypes (such as O157) that have an assortment of impacts on humans which range from harmless to lethal (Maule, 1997; Winfield and Groisman, 2003; Yan and Polk, 2004; Delmas et al., 2015). Specific pathogenic strains cause gastrointestinal disease in humans (Kaper et al., 2004), including Shiga-toxin producing *E. coli* (STEC)—e.g., *E. coli* O157 (Williams et al., 2005; Pennington, 2010). Food-producing animals are sources of pathogenic *E. coli* and may be symptomatic or asymptomatic and, while the infective dose is currently being debated, the general consensus is that a low dose will cause infection (Kirk, 1998; Tuttle et al., 1999; Schmid-Hempel and Frank, 2007). Oporto et al. (2008) determined that 50.8% and 35.9% of sheep and cattle herds, respectively, were carriers of shiga toxin producing strains of pathogenic *E. coli* (Oporto et al., 2008).

Pathogenic *E. coli*, and STEC strains in particular, is a hardy bacterium which can survive for extended periods in water, soil and manure (Maule, 1997; Kirk, 1998; Olson, 2001; Williams et al., 2005; Wiles et al., 2008; Chekabab et al., 2013; Iwu et al., 2021). *E. coli* O157 is sensitive to dehydration/desiccation (Jiang et al., 2002; Williams et al., 2005; Vidovic et al., 2007). *E*. *coli* persistence was shown at 59.1% of chicken litter samples taken from urban poultry farms in Cameroon (Ngogang et al., 2021). Xing et al. (2019) showed that diverse microbial populations reduced the invasive capacity of *E. coli* (van Elsas et al., 2012). In non-autoclaved soils, survival was higher at 5°C than 15°C which may be attributed to the ability of *E. coli* to adapt and survive at lower temperatures and could play a part in survival within soil (Jiang et al., 2002). A study by Yao et al. (2015) focused on *E. coli* O157 survival in microbial active soils in southeast China. They reported a reduction from 10^6^ CFU g^–1^to 100 CFU g^–1^ at a temperature of 25°C in chicken manure amendments after 2.57 ± 6.57 days. Pig manure amendments required 25.65 ± 7.12 days for a similar level of reduction. *E. coli* O157 survival in soil has been shown to increase in soils of higher pH and lower diversity. Studies linking the influence of environmental factors such as soil pH and microbial diversity to *E. coli* survival in soil, in tandem with the influence anthropogenic factors such as manure amendments, could allow for greater transmission across the environment (Jiang et al., 2002; van Elsas et al., 2012; Yao et al., 2015). The high prevalence of *E. coli* in soils highlights the need for proper management of manure resources, particularly prior to spread on agricultural soils (Sobur et al., 2019).

### *Salmonella* spp.

*Salmonella* is a facultative anaerobe with many serotypes capable of causing gastroenteritis in humans. Some serotypes of *Salmonella* can cause infection and fatalities in livestock (e.g., some *Salmonella* Typhimurium and *Salmonella* Choleraesuis strains in pigs) but can also exist as a subclinical infection where no symptoms are detected (Smith et al., 2018). The infective dose is debated throughout the literature, but most agree that the dose required is comparatively higher than for that of *Campylobacter* and *E. coli* (Blaser and Newman, 1982; Kothary and Babu, 2001; Vernozy-Rozand et al., 2002; Hara-Kudo and Takatori, 2011). An increase in the occurrences of antimicrobial resistant strains of *Salmonella* has been reported (Monack, 2012; Williamson et al., 2018), where *S*. Typhimurium has been the most frequently encountered (Glenn et al., 2011).

*Salmonella* survival in soil is dependent on variables such as temperature, predation and introduction method (Jacobsen and Bech, 2012). Guo et al. (2002) reported *Salmonella* transmission to tomatoes from inoculated soils (Guo et al., 2002). Tomatoes placed with cut stem facing topsoil, saturated with sterile water saw population increases of 2.5 log_10_ CFU over an initial 4-day period following inoculation and this remained constant up to 10 days at a storage temperature of 20°C and high humidity. The same study determined that *Salmonella* levels remained constant within the soil over an initial 14-day period with only a small decline over a 45-day period. This suggests that in certain conditions, fruits such as tomatoes exposed to *Salmonella* contaminated soils may be enablers of bacterial population growth/maintenance, as well as a vehicle for transmission to human populations. Elimination of such bacteria prior to manure spreading could greatly reduce this risk. *Salmonella* spp. were observed to invade the flesh of tomatoes (Guo et al., 2002), lettuce and whole green onion (Ge et al., 2012) a process referred to as *Salmonella* internalization—which suggests that typical washing techniques may fail to remove the bacteria and reduce the risk of infection. Ge et al. (2012) determined that weather events have the potential to aid internalization. This highlights the need for area specific studies and legislation as climate and weather events are variable depending on location (Ge et al., 2012). *Salmonella* spp. are reported to survive for prolonged periods outside of a host should environmental conditions be favorable—for example, moisture, temperature, predation and soil type as reviewed by Jacobsen and Bech (2012). Semenov et al. (2009) reported longer survival times of *S. typhimurium* in surface applied manure (estimated 60 days) compared to 10 cm injected slurry (estimated 28.6 days) showing a clear influence of the application method (i.e., surface spreading or injection) and type of material applied (i.e., manure or slurry) (Semenov et al., 2009). Survival of *Salmonella* within the soil is also influenced by the ability to respond rapidly to stress such as entering harsh, non-optimal environments such as soil (Waldner et al., 2012). Spector and Kenyon (2012) explain that by entering the VNCS quickly, the organism allows for survival in a dormant state in under conditions that could otherwise be lethal (Spector and Kenyon, 2012). In both soil and manure-amended soil *S. typhimurium* has been shown to persist longer at lower temperatures. It has been suggested that notably reduced survival rates of *S.* Typhimurium in manure-amended soils at any temperature is related to protozoan predation (Garcia et al., 2010).

### *Providencia* spp.

*Providencia* spp. are Gram negative, rod shaped, and facultative anaerobic bacteria which are part of the family *Enterobacteriaceae* (Beattie et al., 2020; Esperón et al., 2020; Massé et al., 2021). The well-studied species include *P. stuartii*, *P. alcalifaciens*, and, *P. rettgeri* with these bacteria linked to gastroenteritis, Urinary Tract Infections (UTIs) and bacteraemia (Holman et al., 2016; Chuppava et al., 2019; Esperón et al., 2020; Yoshizawa et al., 2020; Congilosi and Aga, 2021). *Providencia* spp. are considered opportunistic (Massé et al., 2021) and often have the greatest impact on those with weakened immune systems such as the elderly, young children and patients whose immune system was compromised by surgery or burns (Blaiotta et al., 2016; Checcucci et al., 2020; Miller et al., 2020). Infections caused by this genus are less common than other bacterial pathogens such as *Salmonella* and *E. coli*, however, they are associated with high mortality rates, particularly in instances where infection of a urinary catheter occurs (Esperón et al., 2020). *P. rettgeri* and *P. stuartii* are frequently found in soil, water and animal hosts (Agga et al., 2020; Esperón et al., 2020). A study by Resende et al. (2014) identified the presence of *Providencia* spp. albeit at low incidence in the investigation of cattle manure following anaerobic digestion (Laconi et al., 2021). Isolation of this bacteria is often unintentional where presumptive positives for other bacteria are determined to be *Providencia* spp. e.g., a report by Blaiotta et al. (2016) found many isolates obtained from cattle manure were in fact *Providencia spp.* as identified by 16S rDNA sequencing and not presumptive species such as *Shigella* (Blaiotta et al., 2016). Information as to their infection of farm animals is limited, however, the genus has been cited as exhibiting high levels of antimicrobial resistance (AMR) (Esperón et al., 2020) and while infection by *Providencia* spp. is rare, there are potential reservoirs of ARGs in the wider environment (Beattie et al., 2020). It would be beneficial for future studies to investigate the prevalence the impact of *Providencia* spp. within manure and soil microbiomes.

### *Listeria* spp.

*Listeria monocytogenes* was isolated from the soil samples in multiple studies indicating the ubiquity of the pathogen in the natural environment and it was detected as being involved in a series of agricultural and food product-associated outbreaks (Falardeau et al., 2018). Its presence in soils is probably related to its increased resistance harsh temperatures and oxidative stress, increased saline concentrations, acid and desiccation stress (Iwu and Okoh, 2020). Soils characteristics such as a high content of molybdenum were reported to carry higher concentrations of *L. monocytogenes* (Liao et al., 2021) suggesting that such conditions can lead to increased surviability. This bacterium can survive for up to 84 days in soils but higher survival rates for up to 295 days were also detected (Welshimer, 1960; Locatelli et al., 2013). Its persistence in soil depends on intrinsic factors such as physiological state, abiotic factors (physical and chemical conditions), precipitation and temperature, and its also highly depenedent on biotic environmental factors such as inhibitory molecules, protozoan grazing and competition for substrate (Vivant et al., 2017).

Understanding of how *Listeria* adapts to different environmental settings is very important, and the adaptation to the new types of matrices necessitates a genetic adaptation (Vivant et al., 2017). In farm environments the causative agent of listeriosis, *L monocytogenes*, was reported to express high resilience to different waste treatments, plus was still present for more than a month in amended soils even without livestock effluents (Hutchison et al., 2005; Moynihan et al., 2015). It has been shown that the bacterium’s adaptability to soil environments can be reduced through the impairment of *Agr*A gene expression, which regulates motility, chemotaxis, transport and metabolism of amino acids (Marinho et al., 2020). Specifically, *Agr*A and σB factors control the general stress response and can cooperatively modulate the *L. monocytogenes* transcriptome to induce a more precise adaptation and an optimal growth and survival rate. Another similar example of genetic adaptation in a soil environment is represented by the two-component systems recently identified as *Lis*RK (Brunhede et al., 2020). This system controls tolerance to several stresses and is important for pathogen growth and survival in sterile and non-sterile soils (Brunhede et al., 2020).

The high virulence potential of *Listeria*, specifically of the strain *monocytogenes*, is attributed to several molecular factors which are associated with multiple phases of the infection. At the onset of infection, genes such as *inl*A, *inl*B, *inl*F, and *inl*J are responsible for adhesion and invasion and are followed by the expression of *hly*, *mpl*, *prf*A, *act*A, and *plc*B genes which are responsible for the growth and spread of the bacterium (Iwu and Okoh, 2020). According to recent findings, such genes are present in isolates collected from irrigation waters and agricultural soils alongside multiple antibiotic-resistant factors such as *aad*A, *tet*A, *tet*B, *tet*C, *sul*I, *sul*II, and *aac*(3)-IIa genes (Iwu and Okoh, 2020).

## Prevalence

The management of pathogen load within livestock manure has been widely recognized as a potential mechanism for the reduction and prevention of outbreaks infection with *E. coli, Salmonella* and *Campylobacter* (Wang et al., 1996; Vernozy-Rozand et al., 2002; Lim et al., 2010; Larramendy and Soloneski, 2016). The importance of good hygiene practices so the manures are safe for use, i.e., allowing for optimum production that does not compromise environmental health has been outlined (Alegbeleye and Sant’Ana, 2020). Manure handling methods vary greatly across agricultural setups depending on the type of livestock housing, amount of manure produced etc. For example, manure produced in NI is handled mainly as slurry (Smith and Williams, 2016) and many studies have identified different management techniques as factors in pathogen load reduction (Table 3). Nicholson et al. (2005) reported 3-month maximum survival times in stored slurries which frequent temperatures over 20°C for *E. coli* O157 and *Salmonella*. These bacteria survived up to 32 days in 7% dry matter dairy slurry, and up to 93 days 2% dry matter dairy manure suggesting that manure with a greater dry matter content reduces pathogen populations at a greater rate with reference to temperatures, moisture content and pH. Data from Nicholson et al. (2005) reported lower maximum survival times of 1 month or less for the same inoculated pathogens in manure composting heaps which commonly reached temperatures above 55°C, suggesting that handling of manure as a solid is a feasible method of pathogen reduction due to higher temperatures being reached. In contrast, a review by Guan and Holley (2003) highlighted potential benefit in treating manures as a liquid where possible, as less time is required to kill pathogens at 25 and 37°C as temperatures are easier to maintain in the liquid state (Guan and Holley, 2003). Storage of manure at 25°C would reduce pathogen loading (e.g., *E. coli* O157, *Salmonella*, and *Campylobacter*) below detectable levels after a period of 90 days. In this context it is difficult to compare data as many variables can influence the survival of pathogens within the manures. For example, Wang et al. (1996) determined that larger populations of inoculated enterohemorrhagic *E. coli* survived longer at both 37 and 22°C, which would infer that in inoculation experiments, larger populations may react and survive differently to smaller levels of initial inoculum used (Wang et al., 1996). This report noted a more rapid decline in population at 37°C which is potentially linked to the desiccation at these prolonged temperature exposures, hence the warmer temperature of 37°C showed higher reduction in population numbers. *E. coli* were seen to survive for up to 8 weeks in the feces at 37°C, where it was possible the survival was sustained by non-uniform dehydration across the feces. Maule (1997) showed a depletion of viable *E. coli* O157 populations in cattle manure from levels of 10^8^ CFU g^–1^ to less than 100 CFU g^–1^ after 9 days at 18°C (Maule, 1997). Kudva et al. (1998) reported better survival *of E. coli* O157 in naturally occurring populations within manure than in artificially inoculated populations within laboratory experiments (Kudva et al., 1998). The same report showed depletion of viable numbers of *E. coli* O157 in cattle slurry to undetectable levels within 5 days at temperatures of 23 and 37°C, whilst a temperature of 4°C saw detectable levels of 10^5^ CFU g^–1^ after 28 days. For most relatable impact, as pathogen survival is highly variable within laboratory and field experiments, studies would benefit from a focus on field experiments with reference to the ultimate location of the manure application rather than to slurry microcosms in the laboratory.

**TABLE 3:** Studies of manure pathogen load survival in agricultural soil.

Sample type	Study location	Detection method	Pathogen	Manure treatment	Maximum survival (days)	References
Inoculated dairy slurry (7% dry matter)	Field study—Nottinghamshire, United Kingdom.	Selective agar plating following selective enrichment culturing method	*E. coli* O157 *Salmonella* spp. *Campylobacter* spp.	On farm storage	32 185 32	Nicholson et al., 2005
Inoculated dairy slurry (2% dry matter)			*E. coli* O157 *Salmonella* spp. *Listeria* spp. *Campylobacter*		93 93 185 32	
Cattle manure added to sterile soil	Laboratory, America.	Direct plating method of selective agar plaiting of soil suspensions.	*E*. *coli* O157	Incubation 21°C 5°C	231 77	Jiang et al., 2002
Chicken manure applied to non-sterile soils	Laboratory, China.,	Direct plating method of selective agar plaiting of soil suspensions.	*E. coli* O157	Incubation 25°C	2.57 ± 6.57 (reduction from 10^6^ to < 100 CFU g^–1^)	Yao et al., 2015
Pig manure applied to non-sterile soils					25.65 ± 7.12 (reduction from 10^6^ to < 100 CFU g^–1^)	
Pig manure	Laboratory, Denmark.	Direct plating method of selective agar plaiting of soil suspensions, qPCR, RT-qPCR	*Campylobacter coli*	Incubation 4°C 15°C 22°C	24 7 6	Bui et al., 2011
Manure amended soils	–		*Salmonella* spp.	–	Up to 332	Jacobsen and Bech, 2012

## Manure as a Vector of Antimicrobial Resistance

The carriage of resistant and pathogenic bacteria, ultimately facilitating the spread of antimicrobial resistance in the environment via animal manure as fertilizers, poses a latent risk for transferring ARGs from animals and animal products to the human microbiome (Zhang et al., 2019). An example of ARG transmission to humans occurred via gene transfer from manured soil to vegetables and was previously observed in lettuce, which underlines the possible risks of transition of plant modified resistome to the human food chain (Zhang et al., 2019). Another illustration of ARG transmission in the food chain was recently identified in fresh vegetables and fruits from southern China, where *Tet* and *ami* genes were present at a very high frequency displaying an average abundance of 3.08 and 1.18 copies per 16S rRNA gene copies (Xiong et al., 2019). In addition, the presence of *aadA*, *aph1*, *floR*, *sul1*, *intI1*, *qacE*, *sul2*, *tetB*, and *tetM* from the food samples displayed the detection frequency above 90%, while *cmlA* and *ermB* depicted slightly lower values.

Several classes of antibiotics are used in food-farm production of which the main are considered tetracyclines which encodes *tet* genes family, sulfonamides encompassed from three types of *sul* genes, β-lactams are represented by *bla*_*TEM*_ and *bla_*CTX*–*M*_* genes, macrolides that carry *erm* and *mef* genes, colistins with different *mcr* genes and quinolones which encodes a family resistant *qnr* genes (Lima et al., 2020). The genes presented in association with animal manure can also attribute resistance to disinfectants in addition to aminoglycosides, tetracyclines, sulfonamides and macrolide-lincosamide-streptogramin B (MLSBs). Resistance genes are frequently linked on mobile genetic elements (MGEs) such as specific plasmids, transposons and insertion sequences/gene cassettes. The latter can capture genetic material from various environmental sites and distribute them amongst the other bacterial species via horizontal gene transfer (Muurinen et al., 2017).

It has been suggested that the analysis of the animal manure and soil resistomes can play a fundamentally important role to underline necessary antimicrobial resistance mechanisms and evaluate the public health risks that might be caused by livestock effluents (Noyes et al., 2016). Moreover, manure-soil microcosms may be reproduced to investigate the spread process of ARGs from animal manure to humans and suggest mechanisms to limit the expansion of ARGs (Wang et al., 2017). First of all, ARG management could focus on eliminating ARGs bearing a low natural reduction potential (e.g., *sul1*, *sul2*, *intl1*, and *tetM*) prior to the manure being applied to the soils. Changing some of the manure handling practices to the novel manure handling methods can potentially reduce the abundance of ARGs in manure utilized for land treatments (Ruuskanen et al., 2016; Gurmessa et al., 2021). Processes such as extending windrow composting and stockpiling have previously been shown to reduce resistance determinants of erythromycin, tetracycline and sulfamethazine between 0.5 and 3 logs in contrast to original cattle manure levels (Xu et al., 2016).

The concentrations of ARGs from livestock waste are found in much higher proportions than the human waste and manured soil, with the reported levels of ARG approximately 28,000 times higher than in un-manured soil (Lima et al., 2020). Within a study, 109 ARGs related to the veterinary and human antibiotic practices were recently identified from fresh poultry, swine and cattle manure of 12 large production-scale Chinese farms (Lima et al., 2020).

Fresh manure application was reported to induce more ARGs in the land than stockpiled manure, which introduces a lesser amount of ARGs (Xu et al., 2021). Previous studies found that the dissipation of ARGs via composting of manure enriched with antibiotics showed distinct differences in contrast to the manure produced by cattle with the dietary inclusion of antimicrobials (Xu et al., 2018). For example, the genes such as *ermX*, *sul1*, *sul2*, and *tetB* were detected in cattle manure after 175 days of post-excretion, which may pose a pollution risk factor of ARGs dissemination if such manure will be further applied into the field. The most prevalent and highly proportional gene set identified in the cattle manure is tetracycline resistance *tetM* and *tetW* genes. During the composting process, the intensity of erythromycin, sulfamethazine and tetracycline resistance gene can be reduced. However, others can remain stable, and prolonged thermophilic composting phases is suggested to decrease the dissemination of ARGs into the encircling environment upon field application of composted manure (Xu et al., 2018).

It is also very important to evaluate persistence studies of ARG from manure soil microcosms in order to understand the environmental antimicrobial resistance status and the shifts in resistance between bacterial communities. A recent study investigated two sets of simulated manure-soil microcosoms by sequencing 16S rDNA to observe bacterial community diversity and directly measuring the fate of ARGs, including *cfr*, *ermB*, *ermF*, *fexA*, *intI1*, *sul1*, *sul2*, *tetB*, and *tetM* and revealed interesting differences in persistence (Wang et al., 2018). Following 90 experimental days, the abundance of ARG from poultry manure showed significant decreasing trends, while the diversity and richness of species in soil were notably increased. The decay rate of *sul1*, *sul2*, *tetM*, and *intI1* within the soil was lower for this set of genes whilst *ermB* and *ermF* genes exhibited increased dissipation rates. Cattle manure contained a more prominent macrolide *ermB* and *sul1* resistant gene set than the poultry litter (Gurmessa et al., 2021). The observation of the wide distribution of *erm* genes was linked with erm proteins accountable for the demethylation of singular adenine base from the 23S rRNA, which is a region that encodes targets for lincosamides, macrolides and streptogramins B that explains the wide-spreading resistance potential of that gene. Indoors to this study, the expression of the *cfr* abundance was weaker expressed (Wang et al., 2018). The *cfr* is generally associated with the plasmid presented in *Staphylococcus* isolates and is collectively expressed with *erm* resistance genes. Similarly, their study reported a decreased *tetB* abundance which is usually connected with the presence of Gram-negative bacteria, compared with *tetM* that is correlated with all types of bacteria. Recently a total of 67 multi-drug methicillin-resistant *Staphylococcus sciuri* were isolated from the 400 environmental samples, of which 40 specimens were from manure, while 360 were from the soil. Strikingly, a major proportion of *S. sciuri* isolates displayed a huge resistance with approximation above 90% against ampicillin, clindamycin, penicillin, cefoxitin and ceftiofur, as well as 86.56% resistance against tetracycline and 50.74% for erythromycin (Kumar et al., 2017). Finally, the study also identified major antibiotic-resistant genes such as *ermA*, *ermB*, *ermC*, *mecA*, *aac*(6′), Ie-aph(2″)Ia, *tetM*, *tetK*, and *mphC* thereby highlighting the dissemination potential of such genes via spreading of swine manure.

In a recent trial that aimed to decrease the pathogen load, the residues of antibiotics and ARGs from poultry manure via composting technique at a field scale were determined and a 10-week intervention was shown to be sufficient to achieve optimized results (Esperón et al., 2020). Firstly, from the sixteen of ARGs, a larger proportion of them declined from the initiation till the end of the experiment by showing remarkable statistically reductions for *tet* genes (A, B, K, M, Q, S, W), *ermB*, *qnrS*, and *bla*_*TEM*_. After 3 weeks of composting, the antibiotic fraction of ciprofloxacin and doxycycline decreased by approximately 90% and reduced the presence of pathogens such as *C. coli* and *E. coli*. Furthermore, the addition of barley straw influenced the OTUs at the phylum level by causing increasing shifts as was noticed in the magnification of the *Flavobacteria* class, which after 1 month was surpassed by the class of *Sphingobacteria* and the composting application modified the bacterial community in favor of the leading order of *Bacillales* (Esperón et al., 2020).

*E. coli* isolated from manure and environmental samples are considerably different from the clinical isolates, which were reported to show resistance patterns against ampicillin, ceftazidime, ceftriaxone, ciprofloxacin, gentamicin, trimethoprim-sulfamethoxazole and levofloxacin (Beattie et al., 2020). Compared to more virulent clinical *E. coli* isolates, those isolated from manure and environmental samples also harbor the plasmids and the potential to develop biofilms responsible for producing a plethora of ARGs (Beattie et al., 2020). A Canadian study isolated *E. coli* from fecal samples from cows, calves and manure pits from dairy farms of Québec. These displayed increased resistance levels toward streptomycin, sulfisoxazole and tetracycline with the main responsible AMR genes *aadA1*, *aadA2*, *aadA5*, aph(3″)-Ib (strA), aph(6)-Id (strB), *tetA*, *tetB*, *sul1*, *sul2* and *sul3* (Massé et al., 2021). The analysis of extended-spectrum β-lactamase from *E*. *coli* samples showed two distinct phenotypes as *AmpC* and ESBL that involved resistance genes *bla_*CMY*–2_* and *bla*_*CTX*–_*_*M*_*. The environmental dissemination of ESBL and *AmpC* producing *E*. *coli* is considered a public health risk factor (Massé et al., 2021). In a German study, approximately 17% of *E. coli* isolates from poultry manure, and excreta exhibited resistance to four antibiotics: Enrofloxacin, Ampicillin, Tetracycline and Sulfamethoxazole-trimethoprim (Chuppava et al., 2019). Antibiotic-resistant patterns were probably attributed to the fattening practices that might be an outcome of intensive farming.

A similar parallel was observed in a recent study for the cattle manure where the fungal genus *Kernia*, along with *Proteobacteria* and *Bacteroidetes*, were enriched from day 0, while enrichment of the *Firmicutes* began at day 99 (Holman et al., 2016). The AMR genes were significantly diminished in concentration over time. The decreases of the 10 out of the 12 antimicrobial resistance determinants and a decline fungal richness by approximately 10-folds compared with bacterial richness was associated with the composting operation by raising the temperature above 55°C (Holman et al., 2016). In a recent trial from Japan, spiked *E. coli* from manure compost was reduced below the detection limit after 55°C incubation within 1 day (Yoshizawa et al., 2020). The study concluded that proper composting could degrade the concentrations of antibiotic-resistant pathogens and ARGs, nevertheless, complete elimination is still difficult to achieve. For instance, physicochemical treatment such as activated carbon with microwave pre-treatment coupled with anaerobic digestion successfully removed several manure contaminants, including ARGs affiliated with macrolides, tetracyclines and sulfonamides (Congilosi and Aga, 2021).

The thermophilic composting process of the poultry, cattle and swine manure can reduce the ARG levels by up to 2.0 logs depending on the type of operational treatment and type of the manure (Checcucci et al., 2020). In another study, swine manure application under 120-day simulated winter incubation decreased the tetracycline resistance genes except for *tetM* and *tetO* genes that persisted in the soil (Miller et al., 2020). Another study concluded that mesophilic anaerobic digestion of the cattle, poultry and swine manure could reduce the proportion of tetracyclines but conversely increase tet and methicillin resistance genes and increase the amounts of total bacteria, *E. coli*, enterococci and *S. aureus*, therefore requiring the need for additional optimizing of the post-digestion operation (Agga et al., 2020). Some of the studies claimed that manure-derived bacteria do not persist in soil for a long time, and manure treatment does not radically affect the soil’s microbiome (Laconi et al., 2021). However, flumequine can likely exhibit selective pressure for *oqxA* and *qnrS* gene accumulation in fertilized soils.

A study based on the investigation of antibiotic-resistant pathogens from chicken litter manure sampled on site from 26 farms in Cameroon has recently detected multidrug-resistant *E. coli* spp. and *Salmonella* spp. isolates (Ngogang et al., 2021). The authors reported that all of the tested *E*. *coli* isolates showed multi-drug resistant patterns, including one isolate resistant to 9 antibiotics out of 11, and 28% of *E. coli* isolated cultures were at least resistant to five antibiotics (Ngogang et al., 2021). In parallel 36% of the *Salmonella* spp. were multi-drug resistant and 27% of the isolates were susceptible to all 11 of the antibiotics. Studies conducted in US farms had earlier reported that *Salmonella* spp. with an increased MDR frequency (58.73%) for at least three or more classes of antibiotics that were isolated from the pig manure application and consequently spread on the land were able to persist for a minimum of 21 days (Pornsukarom and Thakur, 2016). Lately, the same authors found that antimicrobial-resistance plasmids between *Salmonella* serotypes were able to persevere in the farm settings following manure application (Pornsukarom et al., 2017). The authors concluded that *Salmonella* antibiotic-resilient determinants of 95-kb conjugable plasmids showed a distinct transferability amid *Salmonella* serotypes in North Carolina, highlighting the evidence that manure deposition was capable of enriching the environmental resistome.

Several animal manure samples from central Spain revealed specific ARGs such as *aadA*, *tetA*, *tetB*, *str*, *sul1*, and *sul2*. Additionally, the genes from chicken manure included genes with clinical relevance, including *bla_*CTX*–*M*_*, *bla*_*TEM*_, *mecA*, *vanA*, and *qnrB* (Esperón et al., 2018). Meanwhile, the pig slurry samples showed highly expressed *tetC* and *tetM* levels, along with the *mecA* gene. In conclusion, detecting a set comprising 11 ARGs from 18 in amended soil was suggested to support the hypothesis that such a wide magnitude of genes could represent an anthropogenic impact indicator of the environment.

A recent study showed a great prevalence of multi-drug resistant *Enterobacteriaceae*, including their high resistance gene set, observed in the livestock manure that belonged to different farms and slaughterhouses in Portugal (Amador et al., 2019). The authors identified that the most ARGs persistent isolates (79%) were obtained from pig manure that also displayed a resistance rate between 0.6 and 80.8%, and the main ARGs genes were attributed to chloramphenicol (*catI* and *catII*), ciprofloxacin (*qnrS*, *qnrB*, and *oqx*), tetracycline (*tetA* and *tetM*) and trimethoprim/sulfamethoxazole (*dfrIa* and *sul3*), respectively. A similar Enterococcal antibiotic-resistant trait was recently mentioned by other researchers that identified a total of 835 enterococci from soil and chicken litter with the most prevalent species of *E. casseliflavus* and with the least infrequent being *E. gallinarum* (Fatoba et al., 2021). Their results described that approximately half of the isolates showed strong resistance to at least one of the evaluated antibiotics with the highest resilience against tetracycline (33%) at the same time, all of the isolates were susceptible to gentamicin linezolid and tigecycline.

## Conclusion

A complex relationship exists between soil health and agricultural practices. Agriculturally induced soil property alterations have the potential to influence the soil microbiome, directly and indirectly, and subsequently impact on soil health. Application of manure to agricultural land can influence physical, chemical and biological soil properties. Microbiological contents of manure have the potential to spread zoonotic pathogens and ARG’s to the wider environment, where altered pathogen levels in any instance may have a detrimental impact on human and livestock populations. Understanding the relationship between manure management, microbial content, land application, soil and manure microbiome interaction and pathogen survival is key in finding the sensitive and important balance between effective and sustainable agricultural production.

## Author Contributions

ZB, IB, LB, PN, JD, and NC wrote the manuscript. All authors read and approved the final manuscript.

## Conflict of Interest

The authors declare that the research was conducted in the absence of any commercial or financial relationships that could be construed as a potential conflict of interest. The handling editor declared a past co-authorship with one of the authors NC.

## Publisher’s Note

All claims expressed in this article are solely those of the authors and do not necessarily represent those of their affiliated organizations, or those of the publisher, the editors and the reviewers. Any product that may be evaluated in this article, or claim that may be made by its manufacturer, is not guaranteed or endorsed by the publisher.
